# Treatment of an Upper Extremity Chronic Repetitive Strain Injury of 28 Years Duration in a Professional Jazz Saxophonist Using 5% Dextrose

**DOI:** 10.7759/cureus.4116

**Published:** 2019-02-22

**Authors:** Andre Panagos

**Affiliations:** 1 Rehabilitation Medicine, New York University / Langone Health, New York, USA

**Keywords:** fascia, repetitive strain injury, chronic musculoskeletal pain, ultrasound-guided procedures, musculoskeletal ultrasound, musculoskeletal symptoms, pain management, performing arts medicine, musician, neurogenic inflammation

## Abstract

The treatment of chronic repetitive strain injury is a frustrating discourse of potential pathoanatomical causes and their treatments. This case describes an overlooked pathoanatomical cause and successful treatment for a chronic and debilitating repetitive strain injury of the upper extremities that lasted for 28 years and was resistant to a variety of conventional and alternative treatments in a professional jazz saxophone player. A series of fascial tissue infiltrations using 5% dextrose was used to successfully downregulate c-fiber activity within the upper extremities. This treatment resulted in the complete resolution of upper extremity pain and dysfunction with a full return to normal instrument practice and performance that has been sustained without recurrence for four years following treatment.

## Introduction

Repetitive strain injury is caused by repetitive, sustained, and forceful movements [1]. Although mild to moderate cases can resolve with rest and activity modification, this option is not available to professional musicians who replicate precise movement patterns which are intense, forceful, and repetitive. Musicians are extremely focused and dedicated, so they are at very high risk of sustaining repetitive strain injuries since they practice and perform many hours a day and possess a tremendous incentive to continue playing [2]. Musculoskeletal complaints in musicians occur more frequently compared to the general working population, with a lifetime prevalence of up to 93%. Complaints in musicians most commonly occur in the upper extremity and the back [3]. Up to 66% of complaints in musicians in a private clinic were reported as non-specific [4]. Levels of overall job satisfaction are very high, so this limits the scope for psychological interventions, which can be common in the general population [5].

Many theories describe the underlying pathophysiology of repetitive strain injuries and include incomplete repair of local muscle tissue, scarring, tendinosis or injury to local nerve or fascial tissues that make up the musculotendinous unit. Pathological accommodation to persistent tissue damage can occur with changes in afferent sensory information, dystonia, and central sensitization which have an effect on descending neural control of peripheral tissues to protect injured tissues from further injury [1].

The first step in the diagnosis of repetitive strain injury is to rule out more conventional conditions of the upper extremity which include referred pain from cervical spondylosis, radiculitis, shoulder pathology such as labral tears and rotator cuff tears, tenosynovitis in the arm or forearm, peripheral nerve injuries such as a median neuropathy at the wrist, and epicondylitis at the elbow [2]. In these cases, magnetic resonance imaging or electromyography may be helpful, but laboratory tests are frequently of little value unless the symptoms represent a systemic condition such as those found in inflammatory arthritides.

Treatment of repetitive strain injuries can be very frustrating for the professional musician and the clinician. Conventional treatment options include physical therapy, activity modification, bracing, and oral or topical analgesics. The use of corticosteroid injections should be minimized due to their potential to cause further local tissue damage. This case describes the successful use of a 5% dextrose solution to treat pain associated with a repetitive strain injury in a professional musician. This treatment should be included as an option in early as well as refractory cases for musicians who need a timely and permanent solution for pain associated with repetitive strain injuries.

## Case presentation

This case describes the history and course of treatment of a 54-year-old professional jazz saxophonist who presented to a private clinic with a complaint of worsening pain in the left > right trapezius and triceps muscles as well as in the right > left proximal wrist and finger extensors with radiation to the fingers that had been worsening over the course of 28 years. He attributed his pain complaint to the many years he dedicated to perfecting his technique on the jazz saxophone. He described his pain as a fluctuating sharp, heavy, and burning sensation with occasional tingling in the forearms and fingers. He was unable to relate his pain on a visual analog scale so that measurement was deferred. His pain was also associated with loss of finger dexterity which made it difficult for him to play his saxophone for more than 10 minutes at a time. His pain was improved with rest and worsened with any activity requiring the use of his arms. He tried many medications over the years and found dimethyl sulfoxide (DMSO) and topical menthol balms to be the most helpful, although only temporarily.

He underwent many electromyography/nerve conduction studies and imaging studies that he reported as negative. These were conducted internationally and were not available.

He tried many conventional and unconventional treatments over the years including ice, heat, bracing, physical therapy, chiropractic, prolotherapy, osteopathy, craniosacral therapy, structural integration (Rolfing), Alexander technique, yoga, tai chi, meditation, use of Chinese herbs, acupuncture, numerous dietary approaches including a ketogenic diet, and finally voodoo. He reported no surgical interventions.

Past medical history included hypothyroidism which was treated with thyroid replacement and a history of Lyme’s disease that was treated with antibiotics. He had taken many oral medications for his pain but was not taking any at the time of his first visit as he did not find them particularly helpful.

On physical examination, he demonstrated no tenderness at the bilateral medial or lateral epicondylar regions. Characteristic tenderness to palpation was noted at the midportion of the bilateral wrist extensor muscles which corresponded to a thickened deep fascial tissue plane on a brief ultrasound examination. The bilateral nature of the ultrasound findings made it difficult to determine local pathology. Neck range of motion was slightly limited in most planes with no pain. Spurling’s test was negative bilaterally. Bilateral shoulder examinations were unremarkable except for tight bilateral upper trapezius muscles.

We decided to treat the entire diffuse region first with superficial fascial injections using a 5% dextrose solution buffered with 8.4% sodium bicarbonate based on clinical research by John Lyftogt [6]. We first utilized a superficial intradermal approach targeting the Valleix points of the superficial c-fibers of the bilateral dorsal scapular nerves, supraclavicular nerves, axillary nerves, inferior lateral brachial cutaneous nerves, and lateral antebrachial cutaneous nerves. One week after his first treatment he reported a 50% improvement with greatly decreased muscle tension. He was discharged by his osteopath due to the noticeable tissue improvements.

Following the second treatment one week later, he reported an overall 80% improvement and he was able to play his saxophone with his former intensity and speed with minimal discomfort although he felt hesitant to push further. After the third treatment, one week later, he reported a 99% improvement but when he started to play at his former aggressiveness, he noted a new left volar forearm pain that was different from his presenting pain complaints. Following the fourth treatment one week later, he reported a 50% improvement in his new discomfort with pain now localizing only to the bilateral volar forearms. When he played more aggressively and repeated music his pain worsened, but when he varied his music he found he could play very intensely with little pain. Following the next three treatments that were done in one-week intervals, he was able to play normally without accommodations for the first time since the condition began 28 years ago with no pain during, following or several days following a practice set.

As he practiced and performed with ever greater intensity and duration, over the next several weeks he noted a return of his pain localizing to the bilateral trapezius, triceps, and forearms. This pain was treated with ultrasound-guided hydrodissection at each of the sites using the buffered 5% dextrose solution. Following this he was pain-free, and he has remained pain-free for the past four years. He gradually increased his musical output and produced several more CDs before going on a world tour the following year.

## Discussion

This case highlights neurogenic inflammation as an overlooked cause of pain related to chronic repetitive strain injury. The more commonly known classic acute pain complaint is the result of mechanical, thermal or chemical injury and the degree of pain reflects the extent of tissue damage. The primary sensory nerves called A-alpha (proprioception) and A-beta (sensory) nerves send signals to the brain after trauma, fractures, lacerations, pinched nerves and disc herniations [7]. This pathway allows the pain to be identified, rationalized, and localized [8].

Chronic pain is experienced via a different neuronal pathway. Local tissue damage is detected by c-fiber (mechanical, thermal and chemical sensitive) and A-delta (pain and cold temperature sensitive) sensory nerves [7]. The nerve signals are first sent to the nerve roots within the spinal cord where the signals sensitize adjacent nerves resulting in muscle spasms in the regional area or joint [8]. At this level, the pain signal can also be sent to the contralateral side of the body, resulting in the development of a bilateral pain complaint, or they can spread proximally and distally from the original site of injury. The pain signals are then transmitted from the spinal cord to the insula cortex in the brain [8]. The insula cortex is associated with feelings and emotions, so this type of pain is commonly associated with fatigue, anxiety, despair, depression, and fear. This type of pain develops over a period of time after injury, fluctuates in intensity, and is often difficult to localize. This nerve irritation can also cause distal neural elements and tissues to become increasingly sensitive including joints, muscles, and skin which can be equally impacted since they share a common nerve supply. This phenomenon, called Hilton's Law, was first described by John Hilton from his experience treating battlefield injuries in the American Civil War and described in a series of medical lectures given between 1860 and 1862 [9].

C-fibers are the smallest and most abundant nerves in the body. They have unmyelinated axons and have the slowest conduction velocities of any nerve at 0.2–2 m/s. A-delta fibers, in contrast, have medium diameter cell bodies with lightly myelinated axons and slightly greater conduction velocities of 5–30 m/s. C- and A-delta fibers are thought to respond primarily to noxious mechanical, heat, and cold stimuli [10]. As these fibers approach the skin surface, they lose their already thin myelin sheath in the papillary dermis and terminate as free nerve endings in the epidermis, which allows for local treatment [11].

C-fibers are subdivided into peptide-rich (peptidergic) and peptide-poor (non-peptidergic) fibers. Peptidergic c-fibers innervate cutaneous, muscle and joint tissue, whereas non-peptidergic c-fibers only innervate the skin. Both fibers travel in bundles in the upper and lower dermis of the skin, forming a plexus that runs parallel to the dermal-epidermal junction. Peptidergic fibers terminate as free nerve endings in the epidermis and associate with blood vessels, sweat glands and hair follicles [12]. Non-peptidergic fibers terminate as free nerve endings or associate with hair follicles. Compared to their peptidergic counterparts, non-peptidergic fibers have a greater density and distribution in the skin and terminate in more superficial layers [12]. Subcutaneous tissue and the outer layer of the fascia have been found to harbor a high density of these nerve fibers [13]. Hence, the surrounding fascia is much more pain sensitive than adjacent muscle tissue and a likely target for treatment [14]. The c-fibers and A-delta fibers also make up the nervi nervorum of larger nerves which carry sensory nerve impulses in orthodromic and antidromic directions and provide information about metabolic disturbances within the local neural micro-environment as well as support local tissue homeostatic control, which can be another location of treatment.

Over time, chronic pain develops via local and regional inflammation of the neural tissues and recovery may take months or never be complete resulting in persistent abnormal sensory function or neuropathic pain [15]. There is also an observed change in the innervated tissues called the Valleix phenomenon, which describes the development of proximal and distal limb tissue tenderness as well as swelling along the nerve trunk caused by the dorsal root ganglion reflex within the spinal cord [16]. Valleix points describe various points along the course of a nerve in which pressure causes severe local pain (tactile allodynia) as well as shooting pain to other regions (neuralgia). These points are found where the nerve may be structurally compromised such as when the nerve emerges from a bony canal; where it pierces a muscle or fascia structure to reach the skin; where a superficial nerve is easily subject to compression or friction due to adjacent fascia, tendons, ligaments or bone; at a nerve branch point; and where the nerve terminates in the skin [16].

All nerves including nociceptors, have high metabolic activity and require a high capacity energy supply compared with surrounding tissues. C-fibers need a constant supply of glucose to maintain their membrane potential, maintain axoplasmic flow, generate appropriate action potentials, and protect the blood-nerve barrier [17]. Greater signal conduction makes nociceptors highly vulnerable to an oxygen-glucose deprivation injury [18]. As the high capacity energy supply fails, there is an associated increase in c-fiber firing. Using a tourniquet ischemia model, it was found that an oxygen-glucose deprivation injury activates c-fiber nociceptors resulting in pain. C-fiber nociceptors respond to hypoglycemia/glycopenia with an increased firing rate of 652% compared to hypoxia with an increased firing rate of 220% [19]. Next, the increased neurogenic sensory activity results in a reflex endocrine response via the activation of the TRPV1 and TRPA1 transmembrane ion channels in the nerves causing the subsequent release of the pro-inflammatory neuropeptides substance P (SP) and calcitonin gene-related peptide (CGRP) from the peripheral terminal ends which triggers neurogenic inflammation [18]. SP results in an immediate increase in microvascular permeability, attracts immune cells, upregulates the body’s stress response, and impairs local muscle activation [20]. CGRP is involved in immediate vasodilation, tissue degeneration, and calcification. This sensory nerve-mediated axon-reflex results in the characteristic increase in cutaneous vasodilation, which comprises an edematous wheal, local erythema due to increased blood flow, and a skin flare observed surrounding the affected site, as well as an increase in endothelial pressure of neural tissue [20].

Sensory nerves also play a role in the recovery of healthy microcirculation which is very important for wound healing. Substance P and CGRP exhibit a pro-inflammatory role in disease including the activation of neutrophils and eosinophils that promote wound healing. These neuropeptides are also found to maintain skin integrity as the selective depletion of capsaicin-sensitive nerves has been found to result in cutaneous lesions [20]. Proper tissue healing is thought to be the underlying effect that allows the initial use of 5% dextrose to maintain improvement years later and under increased tissue load.

## Conclusions

This case demonstrates neurogenic inflammation from c-fiber pathology as a primary pain generator and the utility in treating these fibers with 5% dextrose in professional musicians with long-standing repetitive strain injuries. This is the first case that reports on the successful treatment of repetitive strain injury in a professional musician that is not only safe but can result in a full return to prior musical form and be lasting. A search of the English literature in PubMed did not yield any articles describing a similar approach for treating repetitive strain injury in professional musicians. It is important to consider c-fiber pathology and neurogenic inflammation in the large differential diagnosis of repetitive strain injury and include the use of 5% dextrose as a potential treatment option in highly motivated professional musicians who have a deep-seated desire to return to their music.
